# *X*-Ray Treatment of Tumours

**Published:** 1903-05-09

**Authors:** 


### Hospital Clinics and Medical Progress.

### X-RAY TREATMENT OF TUMOURS.

In a paper published in the New York *Medical Record*, Dr. Coley sums up the present status of the *x*-ray treatment of malignant tumours. Such conflicting statements have been made as to the efficacy and otherwise of this method of treatment, such false hopes have been raised from time to time in the minds of unfortunate sufferers to be followed by the cruelest disappointments, that it is well indeed to have the opinions of such an authority as Dr. Coley, based as they are on careful observation and extensive inquiry, in order that general practitioners may be guided in the advice they give to patients, who may come to be under their care suffering from cancer which is too extensive, or which for some other reason is beyond the possibility of removal by operation. Dr. Coley remarks at the outset that it is a strange thing, when it is remembered that without doubt malignant disease is on the increase, while our means for combating it have, up to the present, failed to keep pace with this increase, that up to recently no serious attempts have been made in a systematic way, to test the value of the *x*-ray treatment in all varieties of malignant tumours.

As an example of the lengths to which some have gone in their conclusions, Dr. Gilman, of Chicago, is quoted. He says: "I have within the last four years treated over 200 cases (of the various forms of the cancer family), of which I have the results, with a record of recoveries somewhat greater than the average recoveries from typhoid fever in ordinary practice." After making this general statement, without the mention of a single case in which the disease entirely disappeared, and the patient remained well sufficiently long to justify one in regarding it as a cure, he concludes that "we have arrived at a stage of rational treatment which will reduce the mortality of cancer to an insignificant factor." Dr. Coley then sets forth his own personal experiences of the *x*-ray treatment of malignant disease, and gives the details of a large number of illustrative cases. From February, 1902, to January, 1903, he has had under his care, in the General Memorial Hospital, 75 cases of malignant tumours. While it is too early to speak of permanent results in these cases, the immediate results have been sufficiently striking to deserve careful consideration. Of these cases 25 were sarcomata of the various types, 17 round-celled, three spindle-celled, one melanotic round-celled, and in five the variety was doubtful. Of the three patients with spindle-celled sarcoma one showed no improvement, one has not been under treatment long enough to justify an opinion, and one is still under treatment with slight improvement. Of the 17 cases of round-celled sarcoma, all inoperable, four have disappeared entirely, yet in every instance a recurrence has taken place in less than a year. In all of these cases the *x*-ray applications were made four times a week, and continued over a period of many months. In the remaining ones it is noted that in a large proportion there was a temporary improvement followed by renewed activity of growth, and it appears that in some cases while the local improvement was fairly well marked yet metastatic deposits were developing. In a few instances no improvement at all was observed.

One case of Hodgkin's disease, with enlarged glands in both sides of the neck, in the axillæ, and in the groins together with an enlarged spleen occupying the entire left side of the abdomen, was very greatly benefited by *x*-ray treatment. The spleen was reduced to one-third its former size, and the glandular swellings almost disappeared. Blood examinations proved the correctness of the diagnosis in this case. In two other similar cases which have been recorded, and in both of which marked improvement followed the *x*-ray treatment, relapses occurred later.

Dr. Coley has observed 21 cases of breast cancer treated by *x*-rays. In one the growth was primary and not inoperable, but the patient absolutely refused surgical interference. In this case the disease continued to develop in spite of the *x*-ray treatment. In four others the *x*-rays were applied as a prophylactic measure subsequent to operation. The remainder were inoperable cases mostly of recurrent disease. In five instances the recurrent nodules disappeared, but in two of these patients recurrence occurred within a few weeks, while a third developed signs of lung involvement from which she died. Eleven cases of abdominal cancer treated with the *x*-rays are analysed. Two cases of carcinoma of the sigmoid and rectum, five of the uterus, one of the bladder, and three large intra-abdominal sarcomas. In one example where the sigmoid and rectum were involved there was decided improvement in the general symptoms, and several pounds gain in weight, while the tumour diminished in size considerably, but there was little if any diminution of pain. One case of inoperable recurrent cancer of the uterus showed no improvement. In a second case, which was judged to be inoperable, the disease had apparently quite disappeared at the end of three months. The other uterine cases all showed decrease of pain and of the amount of discharge.

With regard to technique, Dr. Coley thinks it is too early to lay down any dogmatic rules. The method is in the early experimental stage. The question of the relative value of the static machine and the coil is still unsettled. In tumours of large size it is probable that a 12-inch coil is better than an 8-inch coil, and a high tube better than a low tube. His own cases were treated with 12-inch coils. There is already one serious result that has come about from the laity having, through the press, obtained exaggerated ideas of the value of this method. Patients with cancer, always with the natural dread of the knife, and prone to try all means first, are seeking *x* ray treatment when the tumours are in the early stages and easily removable. While some few of these cases may possibly be cured by the *x*-rays, probably a larger number will become inoperable before the surgeon is consulted. The treatment itself has its dangers. These are of two kinds. There is the *x*-ray burn due to careless application of the rays or to unusual susceptibility on the part of the patient. Secondly, from too vigorous application of the rays in large and very vascular tumours, the rapid breaking-down or tissue necrosis may lead to the patient developing symptoms of toxæmia which may prove fatal; this has occurred in several instances which have come to Dr. Coley's knowledge. To conclude, he does not believe that it is justifiable to use the method in any but inoperable growths, and chiefly those of external origin. At present there is no convincing evidence that the $x$-rays have much effect upon internal growths.

